# Persistency of Indigenous and Exotic Entomopathogenic Fungi Isolates under Ultraviolet B (UV-B) Irradiation to Enhance Field Application Efficacy and Obtain Sustainable Control of the Red Palm Weevil

**DOI:** 10.3390/insects13010103

**Published:** 2022-01-17

**Authors:** Koko Dwi Sutanto, Mureed Husain, Khawaja Ghulam Rasool, Akhmad Faisal Malik, Wahidah Hazza Al-Qahtani, Abdulrahman Saad Aldawood

**Affiliations:** 1 Department of Plant Protection, College of Food and Agriculture Sciences, King Saud University, P.O. Box 2460, Riyadh 11451, Saudi Arabia; ksutanto@ksu.edu.sa (K.D.S.); gkhawaja@ksu.edu.sa (K.G.R.); aldawood@ksu.edu.sa (A.S.A.); 2 Directorate of Estate Crops Protection, Ministry of Agriculture, Jakarta 12550, Indonesia; akhmadfaisal@pertanian.go.id; 3 Department of Food Sciences and Nutrition, College of Food and Agriculture Sciences, King Saud University, P.O. Box 2460, Riyadh 11451, Saudi Arabia; wahida@ksu.edu.sa

**Keywords:** ultraviolet-B, CFU, *Beauveria bassiana*, *Metarhizium anisopliae*, germination

## Abstract

**Simple Summary:**

Entomopathogenic fungi have the potential to control insect pests However, field application has issues with germination and pathogenicity due to ultraviolet irradiation. The purpose of this study was to investigate the persistence of different local and exotic fungal isolates of *Beauveria bassiana* and *Metarhizium anisopliae* in the laboratory under various ultraviolet exposure times, to obtain a fungal isolate enabling long-term management of red palm weevil *Rhynchophorus ferrugineus* Olivier (Coleoptera: Dryophthoridae), a major pest of palm trees around the globe. After 300 min of ultraviolet radiation, the colony-forming unit of a certain fungal isolate has survived. The persistence of certain fungal isolates to ultraviolet irradiation has shown to be promising. Finding a persistent fungal isolate would be helpful in increasing fungal germination and increase its sustainability against harsh environmental conditions in the field. The overall aim of this research was to obtain sustainable control of the red palm weevil, which has become a major invasive pest in many areas outside its native range.

**Abstract:**

The red palm weevil *Rhynchophorus ferrugineus* Olivier (Coleoptera: Dryophthoridae) has become a key invasive pest and major threat to the palm tree worldwide. Several entomopathogenic fungi are used in insect biological control programs. In the present study, persistency of different local and exotic fungal isolates of *Beauveria bassiana* and *Metarhizium anisopliae* was evaluated under UV-B irradiation with different exposure intervals. Several factors, including ultraviolet (UV) light, significantly decrease germination rate of fungi, as UV penetrates and damages their DNA. Several studies have investigated that UV-resistant conidia germinate better under harsh environmental conditions. Seven local and exotic fungi isolates (“BbSA-1”, “BbSA-2”, “BbSA-3”, “MaSA-1”, “BbIDN-1”, “MaIDN-1”, and “MaIDN-2”) were tested in the current study under UV-B irradiation having different UV exposure times (i.e., 15, 30, 60, 120, 180, 240, and 300 min). The colony-forming unit (CFU) in each isolate was used to calculate the survival rate. Results indicated that survival rate of all the isolates decreased under UV-B irradiation for all exposure times compared to no exposure to UV-B irradiation. The CFU number decreased as the exposure time increased. Fungi isolates “MaSA-1”, “BbSA-1”, “BbSA-2”, “MaIDN-1”, and “MaIDN-2” could persist after 300 min exposure to UV-B, while the remaining isolates, such as “BbIDN-1”, and “BbSA-3”, could not persist after 300 min exposure to UV-B. The ultimate objective of the present research was to explore an ultraviolet-tolerant fungal isolate that might be useful in the field application for the sustainable management of the red palm weevil, which has become a key invasive pest in many regions rather than its native range. Most of the fungus isolates studied in the present work were collected from Saudi Arabia’s Al-Qatif region, where the red palm weevil has infested more than ten thousand trees, worth millions of riyals.

## 1. Introduction

The red palm weevil *Rhynchophorus ferrugineus* Olivier (Coleoptera: Dryophthoridae) is a crucial invasive pest and main danger to the palm species globally. It has damaged more than ten thousand of the date palm trees, worth millions of riyals, in Saudi Arabia’s Al Qatif region [1]. Several entomopathogenic fungi are being utilized in insect biological control programs. Because entomopathogenic fungi are living organisms, they are severely harmed by the harsh environmental conditions in the field, and their efficacy is reduced as a result. The efficacy of microorganisms in the field is influenced by a number of factors, including ultraviolet (UV) irradiation. UV is well-known for its negative impact on the survival of some microbial control agents. Ultraviolet B (UV-B) has proved lethal for several microbial agents, particularly within 300 and 320 nm wavelengths [2]. UV irradiation primarily targets the DNA of the living organisms by reactive oxygen species (ROS), including deoxyribose oxidation and strand breakage, which creates a single oxygen atom (O_2_) and peroxides (H_2_O_2_) [3,4]. The UV negatively affect DNA of several fungi, such as *Beauveria bassiana* Balls, (Hypocreales: Cordycipitaceae) [5], *Verticillium lecanii* Zare and Gams, (Hypocreales: Cordycipitaceae), and *album Aphanocladium* Preuss, (Hypocreales: Nectriaceae) [6]. Ultraviolet A (UV-A) delayed conidia germination of *Metarhizium robertsii* (Metchnikoff) Sorokin (Hypocreales: Clacivipitaceae) and *Metarhizium acridum* Driver and Milner, (Hypocreales: Clacivipitaceae) when grown on PDA medium [7]. The UV-B can reduce conidia viability by >50% [8]. Similarly, the exposure of *Metarhizium anisopliae* (Metchnikoff) Sorokīn, (Hypocreales: Clacivipitaceae), and *B. bassiana* to ultraviolet C (UV-C) for 2 h reduced conidia viability by 85% after 24 h incubation [9]. Similarly, different *Metarhizium* isolates exhibited varying conidia germination when exposed to UV-B irradiation for varying times. Conidia germination started to decline 5 h after exposure and only 5% of the conidia germinated 8 h after exposure compared to control (no UV-B exposure), where germination ranged between 97 and 100% [10].

Microorganisms are highly sensitive to extreme environments containing UV and high temperature [11]. Persistence of *M. anisopliae* (M.08/I05) has been tested under field conditions using an auto-contamination trap containing fungi isolates and 43–59.25% decrease in colony-forming unit (CFU) was recorded under 18–30 °C temperature and 60% relative humidity (RH) [12]. However, exposure of *B. bassiana* and *M. anisopliae* conidia to UV irradiation increased their viability [13]. Fungus isolates attained maximum pathogenicity against European corn borers *Ostrinia nubilalis* (Hübner) (Lepidoptera: Crambidae) following exposure to UV light (wavelength 254 nm for 45 min), causing 100% larval mortality within 10 days [14]. Selective *B. bassiana* isolates resistant to UV irradiation have shown good performance that increased their effectiveness against the coffee borer beetle *Hypothenemus hampei* (Ferrari) (Coleoptera: Curculionidae) [15].

Different local and exotic fungi isolates of *B. bassiana* and *M. anisopliae* were tested for their persistence under different exposure times to UV-B in the laboratory. Finding a persistent fungal isolate would be helpful in increasing fungal germination and increase its sustainability against harsh environmental conditions in the field. The ultimate goal of this study was to find ultraviolet-tolerant fungal isolates that might be used in the field for the long-term management of the red palm weevil, which has become a major invasive pest in many areas outside of its natural habitat.

## 2. Materials and Methods

### 2.1. Entomopathogenic Fungi Source

Different indigenous isolates of *B. bassiana* and *M. anisopliae* (originally from Al-Qatif governorate and Riyadh region) were obtained either from the Ministry of Agriculture or collected by the first author from Saudi Arabia. The exotic isolates used in the study were collected from Kalimantan, Indonesia. All isolates were assigned a code based on their origin (SA for Saudi Arabia) and species (Bb for *B.* *bassiana*). The *B.* *bassiana* isolates were coded as “BbSA-1”, “BbSA-2”, and “BbSA-3”. The *M. anisopliae* was coded as Ma and the isolate code was “MaSA-1”. Similarly, Indonesian isolate (IDN) of *B. bassiana* was coded as “BbIDN-1”, whereas those belonging to *M. anisopliae* were coded as “MaIDN-1” and “MaIDN-2”. An identification key was used to identify the isolates based on morphological characteristics [16]. All these fungal isolates were previously pathogenicity tested against red palm weevil *Rhynchophorus ferrugineus* Olivier (Coleoptera: Dryophthoridae) stages in our laboratory [17]. Detailed information regarding the source of fungal isolates used in the study is given in Table 1. All isolates were cultured at the Economic Entomology Research Unit (EERU), Plant Protection Department, College of Food and Agriculture Sciences, King Saud University, Riyadh, Kingdom of Saudi Arabia (KSA).

### 2.2. Isolation and Culture of Fungal Isolates on Potato Dextrose Agar Medium

The body surface of the red palm weevil adults (cadavers) infected with fungi was sterilized with 5% sodium hypochlorite for 1 min and washed twice with sterile distilled water [18]. After washing, a small part of the cadaver was dried on filter paper before transferring to potato dextrose agar (PDA) (Scharlau, Microbiology 01–483, Barcelona, Spain) medium. Fungi were allowed to grow on PDA medium at 85 ± 5% relative humidity and 25 ± 2 °C temperature for 7 days [17]. A hyphen tip technique was used for the purification of fungi [19].

A total 30 g PDA powder was thoroughly mixed with 1 l distilled water and autoclaved at 121 °C for 20 min (sterilization). Autoclaved liquid medium was poured onto sterilized Petri dishes (10 cm in diameter) as a thin layer. After solidification of the PDA, 1 cm of each culture isolate was taken with a sterile scalpel and transferred to the center of the PDA plate. The inoculated plates were incubated at 25 ± 2 °C temperature and 85 ± 5% relative humidity in an incubator (Orbital Incubator, Stuart, SI 500). Initially, the first purification of the isolates was grown on PDA medium, followed by screening of the isolates that germinated rapidly. The purification was performed 15 times through PDA medium to enhance the performance of the isolates [17]. Conidia were produced from an isolated fungus that was cultivated on PDA medium plates for 14 days, after which each plate was supplemented with 10 mL sterile distilled water mixed with Triton-X (0.1%) and then harvested by scraping the inoculum [20]. The concentration of conidia was measured by adding 10 µL of conidial solution to hemocytometer (Improved Neubauer, Germany), and then the total numbers of conidia in zones A, B, C, D, and E were counted under the microscope [21], followed by the calculation of the fungi concentration at 1 × 10^9^ conidia/mL as used for UV testing.

### 2.3. Sterilization and Disinfection Procedure

Sterilization and disinfection procedures were applied to eliminate or kill all forms of microbial agents on the surface of the workplace. All equipment was sterilized prior to use in the experiments. Erlenmeyer flasks, glass beakers, glass tubes, micro pipettes, and pipette tips were sterilized in an autoclave (HIRAYAMA, HG-80, and Japan). Moreover, the workplace was sterilized by alcohol (96%) before and after the experiments to avoid contamination.

### 2.4. Evaluation of the Persistence of Fungi Isolates under UV-B Exposures

Laboratory screening of the most virulent isolates was carried out under ultraviolet B (UV-B) irradiation (280–320 nm wavelength) [6]. Fungi isolates were tested under different UV exposure times (15, 30, 60, 120, 180, 240, and 300 min), and a control 0 min (no irradiation) was included in the study for comparison. Two UV-B lamps (15 watts, 302 nm Fotodyne Inc., New Berlin, Wisconsin, Germany) were used in this study. The lamp tubes were placed horizontally above the fungi samples in the irradiation chamber (60 × 80 cm, ADECO, Riyadh, Saudi Arabia). The samples were kept 20 cm away from the UV-B irradiation light.

### 2.5. Colony-Forming Unit

The colony-forming unit (CFU) was evaluated for each isolate included in the study. The final concentration was 1 × 10^9^ conidia/mL and the fungi suspension was mixed with Tween 80 surfactant (0.001%). Afterwards 1 mL of the solution was poured into small plastic cups (d: 5 cm; h: 3 cm) using a micropipette (1000 mL) to give a thin layer of CFU. The samples were exposed directly to the UV-B light [6]. Each fungus suspension was adjusted to 1 mL (the original volume) after exposure to UV irradiation with distilled water. The suspension was then dispersed on the PDA medium layer in Petri dishes. After the UV exposure procedure, plates were covered with aluminum foil and kept in an incubator (Orbital Incubator, Stuart SI500) under 25 ± 1 °C temperature and 85 ± 5% relative humidity in dark conditions [6]. The dishes were observed daily over 3 days until the CFU in the control were fully grown on PDA media. CFU was used to calculate the survival rate after UV irradiation in most of the UV studies in fungus tolerance [9,10,11]. The CFU were counted by observing the relative percentage of the culture which was measured using an equation given as below.

Relative culturability (%) = (Mt/Mc) × 100(1)

Here Mt is the mean number of CFU of the three replicates at exposure time t, and Mc is the mean number of CFU for all control dishes [6].

### 2.6. Statistical Analysis

The experiment was laid out according to completely randomized design (CRD). Numbers of CFU were calculated from survival percentage of fungi. Relative culturability was estimated for all isolates grown on PDA media. Ultraviolet irradiations were considered as main effects and non-germinated individual conidia as a response variable. The results were analyzed by analysis of variance (ANOVA). The means were compared using least significant difference (LSD) test with threshold of *p* ˂ 0.05 [22].

## 3. Results

### 3.1. Evaluation of the Persistence of Fungi Isolates under UV-B Exposures

The results revealed that all tested isolates were affected by UV-B irradiation with varying CFU survival rates (Table 2). The number of CFU significantly differed after exposure to UV-B for different times for the tested isolates, i.e., “BbSA-1” (F: 69.8; df: 7, 16; *p* < 0.0001), “BbSA-2” (F: 129.7; df: 7, 16; *p* < 0.0001), “BbSA-3” (F: 44; df: 7, 16; *p* < 0.0001), “BbIDN-1” (F: 346.1; df: 7, 16; *p* < 0.0001), “MaSA-1” (F: 66.8; df: 7, 16; *p* < 0.0001), “MaIDN-1” (F: 31.4; df: 7, 16; *p* < 0.0001), and “MaIDN-2” (F: 63.9; df: 7, 16; *p* < 0.0001). The mean number of CFU after exposure to UV-B irradiation for 300 min was 29.2 for “MaSA-1”, 25.7 for “MaIDN-2”, 11.6 for “BbSA-1”, 11 for “MaIDN-1”, 10 for “BbSA-2”, 0 for “BbSA-3”, and 0 for “BbIDN-1”. The isolates “BbSA-3” and “BbIDN-1” were highly sensitive to all UV-B exposure times. The number of CFU in each isolate decreased with increasing UV-B exposure time.

**Figure 1:**
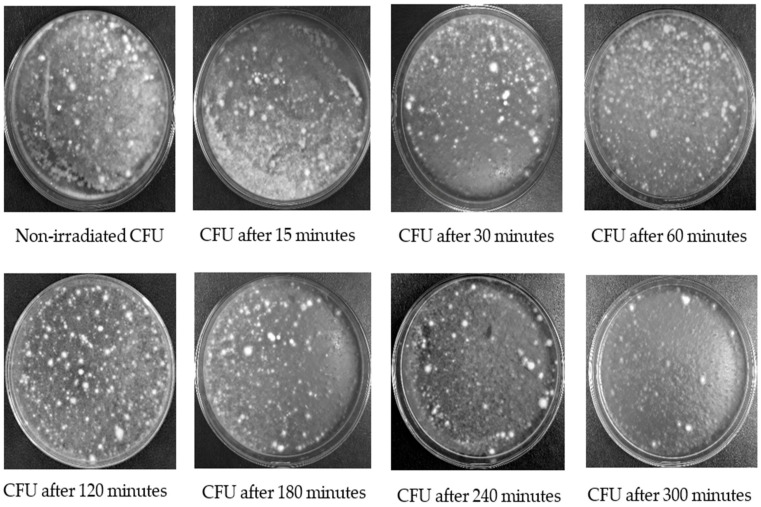
A comparison for CFU of the fungus isolate “MaSA-1” growth on PDA medium after UV-B irradiation at different exposure time.

The surviving CFU after UV-B irradiation can germinate on PDA medium in the Petri dishes. The number of CFU of all isolates decreased after exposure to UV-B irradiation for 15–300 min compared to control treatment (no exposure to UV-B) (Figure 1).

### 3.2. Relative Culturability of Fungi Isolates after Ultraviolet B (UV-B) Irradiation

**Figure 2:**
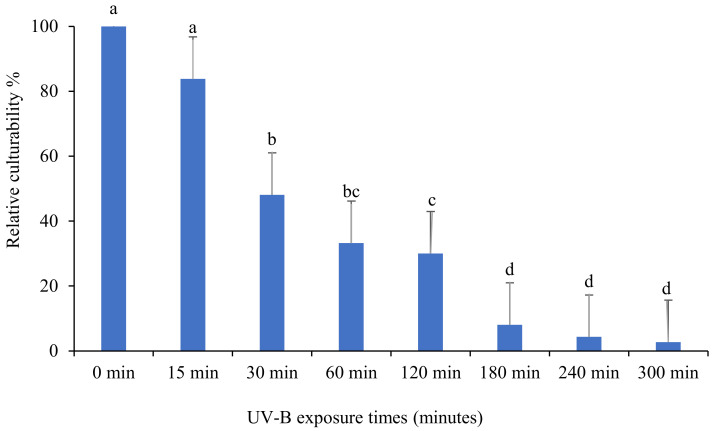
The impact of different UV-B exposure times on relative culturability percentage of *Beauveria bassiana* isolate “BbSA-1”. The mean number of relative culturability percentage ± SE is shown. Different letters on the same patterned bars indicate significant differences (LSD test, *p* < 0.05).

The effect of UV on the culturability percentage of *Beauveria bassiana* isolate “BbSA-1” is shown in Figure 2. Based on the mean number of CFU after growing on PDA medium, the culturability % of *B. bassiana* isolate “BbSA-1” decreased significantly for all exposure durations (F: 36.7; df: 7, 16; *p* < 0.0001). The culturability percentage decreased as exposure time increased, indicating that UV-B was influencing it in a direct proportionate manner.

**Figure 3:**
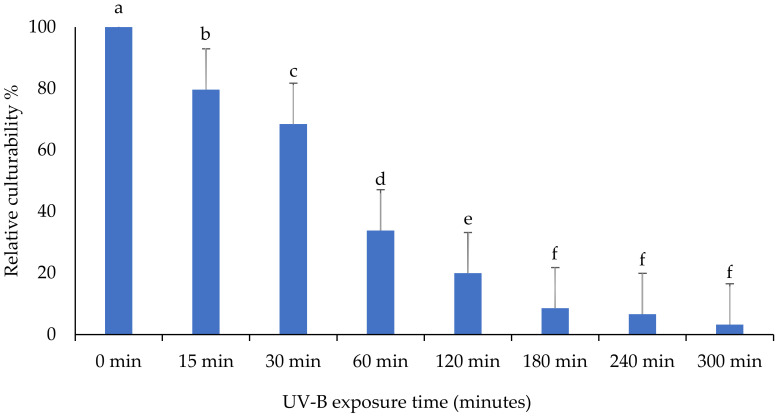
The impact of different UV-B exposure times on relative culturability percentage of *Beauveria bassiana* isolate “BbSA-2”. The mean number of relative culturability percentage ± SE is shown. Different letters on the same patterned bars indicate significant differences (LSD test, *p* < 0.05).

The impact of ultraviolet light on the culturability of the *B. bassiana* isolate “BbSA-2” is shown in Figure 3. The percentage of culturability was significantly different in response to different exposure times for “BbSA-2” (F: 129.4; df: 7, 16; *p* < 0.0001). After 300 min of UV-B exposure, the CFU of this isolate has decreased after growing on PDA medium, but it still persists.

**Figure 4:**
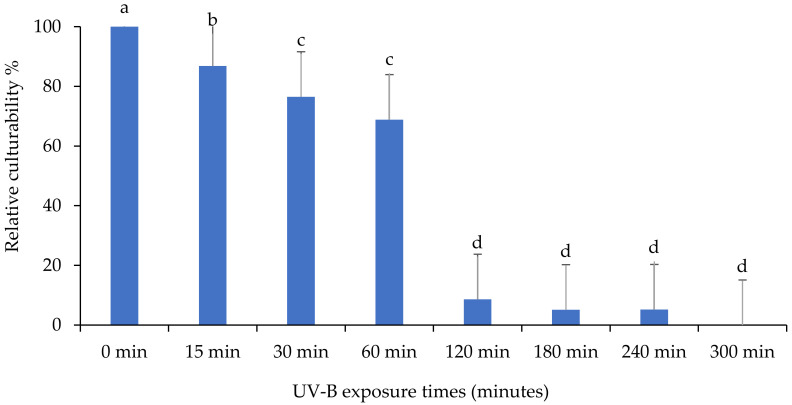
The impact of different UV-B exposure times on relative culturability percentage of *Beauveria bassiana* isolate “BbSA-3”. The mean number of relative culturability percentage ± SE is shown. Different letters on the same patterned bars indicate significant differences (LSD test, *p* < 0.05).

After UV-B irradiation, the culturability percentage of *B. bassiana* isolate “BbSA-3” is presented in Figure 4. Similar to other isolates, there was a significant decrease in the culturability percentage response of “BbSA-3” isolate at different exposure intervals (F: 62.9; df: 7, 16; *p* < 0.0001). The percentage of culturability decreased as exposure time increased, but CFU decreased after growing on PDA medium and did not persist after 300 min of UV-B exposure.

**Figure 5:**
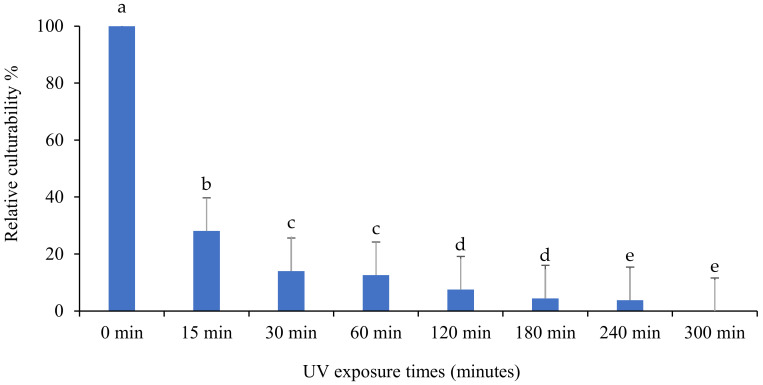
The impact of different UV-B exposure times on relative culturability percentage of *Beauveria bassiana* isolate “BbIDN-1”. The mean number of relative culturability percentage ± SE is shown. Different letters on the same patterned bars indicate significant differences (LSD test, *p* < 0.05).

The effect of UV light on the culturability of the *B. bassiana* isolate “BbIDN-1” is shown in Figure 5. For “BbIDN-1”, the percentage of culturability showed significant differences in response at different exposure times (F: 671.6; df: 7, 16; *p* < 0.0001). With increasing exposure time, the percentage of culturability decreased, but CFU did not persist after 300 min of UV-B exposure. In comparison to other *B. bassiana* isolates, this isolate is highly sensitive to UV-B irradiation.

**Figure 6:**
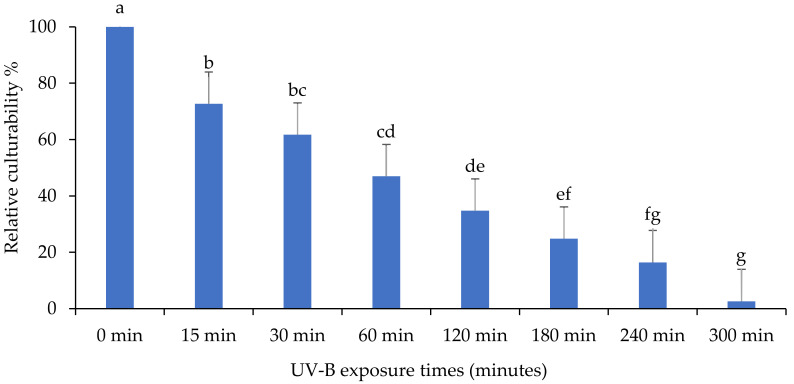
The impact of different UV-B exposure times on relative culturability percentage of *Metarhizium anisopliae* isolate “MaIDN-1”. The mean number of relative culturability percentage ± SE is shown. Different letters on the same patterned bars indicate significant differences (LSD test, *p* < 0.05).

The culturability percentage of *Metarhizium anisopliae* isolate “MaIDN-1” is presented in Figure 6. A significant difference in the percentage of culturability in response to different exposure times was observed for “MaIDN-1” (F: 38.9; df: 7, 16; *p* < 0.0001). The number of CFU was decreased after growing on PDA medium, but CFU still persisted after 300 min UV-B exposure.

**Figure 7:**
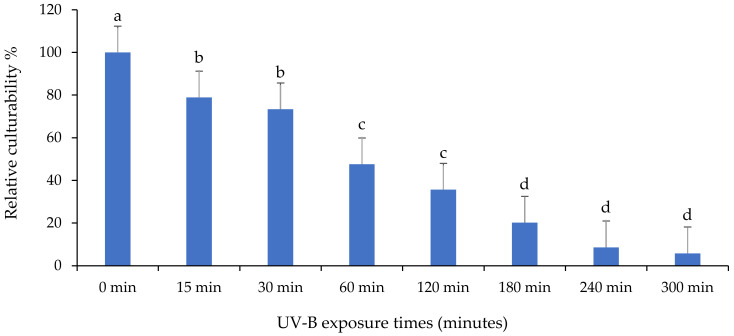
The impact of different UV-B exposure times on relative culturability percentage of *Metarhizium anisopliae* isolate “MaIDN-2”. The mean number of relative culturability percentage ± SE is shown. Different letters on the same patterned bars indicate significant differences (LSD test, *p* < 0.05).

The effect of UV on the culturability percentage of *M. anisopliae* isolate “MaIDN-2” is presented in Figure 7. For “MaIDN-2”, there was a significant difference in the percentage of culturability in response to different exposure times (F: 51.8; df: 7, 16; *p* < 0.0001). Similarly, the number of CFU was decreased, however, CFU still remained after 300 min of UV-B exposure.

**Figure 8:**
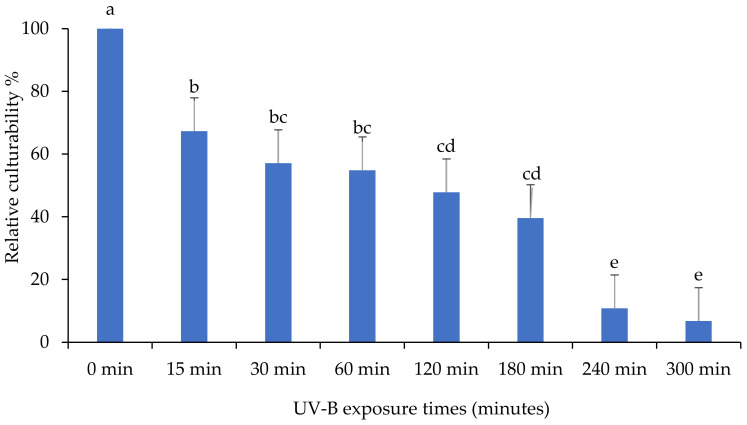
The impact of different UV-B exposure times on relative culturability percentage of *Metarhizium anisopliae* isolate “MaSA-1”. The mean number of relative culturability percentage ± SE is shown. Different letters on the same patterned bars indicate significant differences (LSD test, *p* < 0.05).

The impact of ultraviolet light on the culturability of the *M. anisopliae* isolate “MaSA-1” is shown in Figure 8. The culturability percentage of *M. anisopliae* isolate decreased significantly for all exposure durations based on the mean number of CFU after growing on PDA medium “MaSA-1” (F: 46.1; df: 7, 16; *p* < 0.0001). Similar to other isolates, the number of CFU decreased, but CFU remained after 300 min of UV-B exposure, indicating that this isolate is persistent.

## 4. Discussion

The current study indicated that the number of CFU of each isolate decreased after exposure to UV-B irradiation for different times. UV-B rays had a negative effect on the germination rate of conidia of each isolate included in the study. The result confirmed that UV-B and UV-C are the most destructive rays for fungi isolates [5]. It has been reported that UV-B irradiation affects all fungi isolates and reduces their CFU number [5,6,23]. Reactive oxidation species (ROS), such as single oxygen and peroxidase, are generated after exposure of microorganisms to UV light, which have a negative effect on their DNA [3]. Moreover, biological systems of microorganisms absorb photochemicals when exposed to UV light [24]. The UV light (UV-B) caused the death of *M. anisopliae* conidia and inhibited their germination [25]. In this study, UV-B irradiation affected the conidia germination of fungal isolates included in the study. However, conidial dormancy has been regarded as the key characteristic for most of UV-tolerant fungi isolates [26]. In the present study, the viability of each fungus isolate was determined by the germination of conidia after UV irradiation.

In our study conidia germinated 2–3 days after incubation. This can be supported by another study reporting that CFU of fungi typically remain viable within 2–5 days after exposure to UV irradiation and it greatly depends on the cell type exposed to the irradiation [5]. The CFU of *M. anisopliae* was observed when irradiated to UV-C for 50 s and CFU decreased to 1.11 × 10^8^ conidia/mL, while the CFU under non-irradiated conditions were 11.13 × 10^8^ conidia/mL [27]. Our study determined which fungi isolates can survive after exposure to UV-B irradiation. The survival rate based on the number of CFU of fungi isolates determines conidia germination [6,27,28,29].

Ultraviolet irradiation changed Cyclobutane pyrimidine dimer (CPD) formation in DNA resulting in decreased conidia viability of *B. bassiana* [5]. Although UV irradiation-induced DNA damage chemically alters DNA components, DNA can be repaired by removing site lesions [30]. Physical protection (such as pigmented fungi) could increase tolerance and survival of *M. anisopliae* against UV [31]. However, UV-B exposure decreased viability and pathogenicity of *M. anisopliae* [20], and delayed germination [14,28]. In the present study fungi isolates “BbSA-3” and “BbIDN-1” lost their activity (germination) faster than the isolates “MaSA-1”, “BbSA-1”, “MaIDN-1”, “MaIDN-2”, and “BbSA-2”. In addition, CFU values of fungi isolates decreased after exposure to UV-B irradiation. The highest number of CFU after 300 min UV-B exposure were 29.2 recorded for the isolate “MaSA-1”. This might be linked to the high concentration of fungi isolates that protected it against UV-B irradiation. Similarly, the high concentration could protect *B. bassiana* from UV irradiation [32].

Several studies have investigated the effect of UV irradiation on germination of different fungi isolates. The germination rate of *M. anisopliae* decreased by 50% after 1 h exposure to UV-B irradiation, while 3% of conidia remained viable after 12 h exposure [33]. Similarly, reduction in conidia germination after 5 min of exposure to UV-B was 94% to 52% and 96% to 54% for *B. bassiana* and *M. anisopliae,* respectively [34]. *B. bassiana* and *M. anisopliae* conidia were significantly affected when they were exposed to UV-B for 1–2 h [35]. It has been reported that dried entomopathogenic fungi such as *M. anisopliae* was the most tolerant to UV-B irradiation compared to *B. bassiana* when exposed to UV irradiation for 4 h [36].

However, *M. acridum* isolate proved tolerant to UV-B after exposure to white light (visible light) for 2 h based on germination rate [37]. *M. anisopliae* and *P. fumoroseus*, (Eurotiales: Trichocomaceae) exhibited varied tolerance to UV-B irradiation [38]. Several local isolates of *B. bassiana* and *M. anisopliae* have been tested under UV-B irradiation and *B. bassiana* proved more tolerant than *M. anisopliae*. Nonetheless, *M. anisopliae* tolerated high temperature (37 °C) [39]. A review study showed varied susceptibility of fungi isolates to UV irradiation [11]. The survival rate of *M. anisopliae* conidia was 0% to 1% after 8 h exposure to UV-B, while *M. flavoviridae*, (Hypocreales: Clavicipitaceae) had 0% to 11% survival, indicating that *M. flavoviridae* was more tolerant to simulated sunlight [40]. The sensitivity of several UV-B isolates varies depending on culture age. For example, *M. anisopliae* and *B. bassiana* proved more tolerant to UV-B irradiation when the culture was air dried for 14 days compared to 7 days [13]. We conclude that “MaSA-1” isolate can be selected for further field testing based on its high survival rate under UV exposure in the laboratory.

In an additional case study, fungi survival after UV exposure resulted in increased germination and pathogenicity [28]. Isolates of *M. anisopliae* responded differently to UV-B irradiation; however, surviving conidia were the most virulent to grasshoppers *Schistocerca gregaria*, (Orthoptera: Acrididae) after being exposed to UV for 8 h, and their germination rates ranged from 79.4% to 91.6% [41]. In contrast, UV irradiation reduced the vegetative growth and conidial sporulation of *B. bassiana* strains after successive passages of coleopteran host, i.e., lesser mealworm *Alphitobius diaperinus* (Panzer) (Coleoptera: Tenebrionidae) [42]. The germination and infection of fungal isolates *B. bassiana* and *M. anisopliae* were evaluated on red palm weevil *Rhynchophorus ferrugineus* Olivier (Coleoptera: Dryophthoridae) in laboratory condition, and exhibited more efficient control, but the germination of both fungal isolates was sharply reduced when exposed to sunlight condition at different seasons [12].

Moreover, the use of several microbial agents to control red palm weevil have been well documented [43]. For example, *B. bassiana* and *M. anisopliae* have been reported to be a key determinant in suppressing *R. ferrugineus* populations in the field [44]. This may explain why entomopathogenic fungi are the best microbial agents for controlling this pest in the field. However, the pathogenicity of various fungi isolates has recently been tested in the laboratory against red palm weevil stages [17]. When compared to other microbial agents, such as the nematode genus *Steinernema* and *Heterorhabditis*, it showed that different nematode isolates had different mortality responses, especially in the red palm weevil larvae and adult stages [45]. Another study showed that using a combination of fungus, *M. anisopliae*, and nematode, *Heterorhabditis* sp. (Rhabditida: Heterorhabditidae) to control coconut beetle larvae *Oryctes rhinoceros*, (Coleoptera: Scarabaeidae) had a positive result, with high mortality [46].

However, in this study, the selection of fungi isolates is quite important and could be supported in order to achieve long-term performance when applied in the field, as we know that fungi isolates exhibit varying response to UV irradiation based on the association between geographical origin [47], conidial pigmentation [31,48], and host insects [39].

## 5. Conclusions

The complete inactivation of conidia belonging to the isolates “BbSA-3” and “BbIDN-1” was recorded at a concentration of 1 × 10^9^ conidia/mL, while other isolates, i.e., “MaSA-1”, “BbSA-1”, “MaIDN-1”, “MaIDN-2”, and “BbSA-2”, persisted after 300 min exposure to UV-B irradiation. Our findings reveal that the surviving fungi isolates after UV exposures may possess increased germination performance. The surviving isolates should be tested under field conditions for making general recommendations and to achieve the overall goal of this work, to obtain sustainable control of the red palm weevil, which has become a major invasive pest in many areas outside its native habitat. Furthermore, the survival fungus should be tested with various delivery systems and in various seasons to determine its efficacy in the field.

## Figures and Tables

**Table 1:** Background information on the entomopathogenic fungal isolates used in the present study.

Fungi	Isolates’ Code	Insect Species/ Order	Source of Isolation	Original Location (s) of Isolates	Coordinates
*B. bassiana*	BbSA-1	Red palm weevil *R. ferrugineus*/Coleoptera	adult	Al Qatif, Saudi Arabia	N: 26.34437°; E: 43.69217°
*B. bassiana*	BbSA-2	Cotton leafworm *Spodoptera litura* /Lepidoptera	larva	Al Qatif, Saudi Arabia	N: 24.41867°; E: 46.65408°
*B. bassiana*	BbSA-3	Red palm weevil *R. ferrugineus*/Coleoptera	adult	Al Qatif, Saudi Arabia	N: 26.35231°; E: 43.71789°
*B. bassiana*	BbIDN-1	Corn earworm *Helicoverpa zea* /Lepidoptera	larva	Kalimantan, Indonesia	N: −0.02633°; E: 109.3425°
*M. anisopliae*	MaSA-1	Red palm weevil *R. ferrugineus* /Coleoptera	adult	Riyadh, Saudi Arabia	N: 24.41867°; E: 46.65408°
*M. anisopliae*	MaIDN-1	Coconut Rhinoceros beetle *Oryctes rhinoceros*/Coleoptera	adult	Kalimantan, Indonesia	N: −0.02633°; E: 109.3425°
*M. anisopliae*	MaIDN-2	Coconut leaf beetle *Brontispa longissima*/Coleoptera	adult	Kalimantan, Indonesia	N: −0.03962°; E: 109.3128°

**Table 2:** The effect of different UV-B irradiation exposure times on fungi isolate persistency based on the number of colony-forming units (CFU).

Isolate Name	Isolate Code	Colony-Forming Unit (CFU)/Ml of Fungus Isolate							
		UV-B Exposure Times							
		0 Min	15 Min	30 Min	60 Min	120 Min	180 Min	240 Min	300 Min
*B. bassiana*	BbSA-1	433 ± 30 ^a^	355 ± 32 ^b^	203 ± 26 ^c^	143 ± 7 ^d^	127 ± 10 ^d^	33 ± 5 ^e^	18 ± 4 ^e^	11.6 ± 3 ^e^
	BbSA-2	311 ± 5 ^a^	247 ± 16 ^b^	212 ± 17 ^c^	105 ± 14 ^d^	62 ± 3 ^e^	26 ± 1 ^f^	20 ± 3 ^f^	10 ± 1 ^f^
	BbSA-3	368 ± 30 ^a^	323 ± 45 ^ab^	275 ± 23 ^bc^	225 ± 28 ^c^	31 ± 4 ^d^	19 ± 3 ^d^	20 ± 2^d^	0 ± 0 ^d^
	BbIDN-1	340 ± 13 ^a^	95 ± 10 ^b^	48 ± 3 ^c^	43 ± 7 ^cd^	25 ± 2 ^de^	15 ± 2 ^ef^	13 ± 0.1 ^ef^	0 ± 0 ^f^
*M. anisopliae*	MaIDN-1	420 ± 30 ^a^	305 ± 20 ^b^	259 ± 10 ^bc^	197 ± 36 ^cd^	146 ± 2 ^de^	104 ± 25 ^ef^	69 ± 9 ^fg^	11 ± 0.5 ^g^
	MaIDN-2	453 ± 29 ^a^	355 ± 31 ^b^	328 ± 17 ^b^	213 ± 8 ^c^	162 ± 26 ^c^	89 ± 7 ^d^	39 ± 8 ^de^	25 ± 3 ^e^
	MaSA-1	439 ± 24 ^a^	293 ± 9 ^b^	250 ± 10 ^bc^	238 ± 13 ^c^	207 ± 14 ^cd^	171 ± 23 ^d^	47 ± 6 ^e^	29 ± 4 ^e^

The mean number of colony forming units (CFU) ± SE is shown. Within each row, means followed by the same letters do not differ significantly (LSD test at *p* < 0.05).

## Data Availability

All relevant data are within the paper.
